# Short-term echocardiographic follow-up after hospitalization for COVID-19: a focus on early post-acute changes

**DOI:** 10.3389/fcvm.2023.1250656

**Published:** 2023-11-22

**Authors:** Oleksii Honchar, Tetiana Ashcheulova

**Affiliations:** Department of Propedeutics of Internal Medicine, Nursing and Bioethics, Kharkiv National Medical University, Kharkiv, Ukraine

**Keywords:** COVID-19, long COVID, echocardiography, cardiac remodeling, cardiac dysfunction, hospitalization, convalescence

## Abstract

**Background:**

Impaired physical functional status is one of the typical long-term sequelae of COVID-19 infection that significantly affects the quality of life and work capacity. Minor changes in cardiac structure and function that are unable to cause the manifestation of overt heart failure may remain undetected in COVID-19 convalescents, at the same time potentially contributing to the persistence of symptoms and development of long COVID syndrome.

**Purpose:**

To study the typical features and short-term dynamics of cardiac remodeling and possible signs of cardiac dysfunction following hospitalization for COVID-19.

**Methods:**

This is a combined cross-sectional and longitudinal cohort study in which 176 hospitalized patients (93 female and 83 male, mean age 53.4 ± 13.6 years) with COVID-19 infection underwent comprehensive transthoracic echocardiography pre-discharge (22.6 ± 7.1 days from the onset of symptoms) with repeated evaluation after 1 month. The control group included 88 age-, sex-, height- and weight-matched healthy individuals, with a subset of those (*n* = 53) matched to the subset of non-hypertensive study participants (*n* = 106).

**Results:**

Concentric left ventricular geometry was revealed in 59% of participants, including 43% of non-hypertensive subjects; predominantly Grade I diastolic dysfunction was found in 35 and 25% of patients, respectively. Other findings were naturally following from described phenotype of the left venticle and included a mild increase in the absolute and relative wall thickness (0.45 ± 0.07 vs. 0.39 ± 0.04, *p* < 0.001), worsening of diastolic indices (e’ velocity 9.2 ± 2.2 vs. 11.3 ± 2.6 cm/s, *p* < 0.001, E/e’ ratio 7.5 ± 1.8 vs. 6.8 ± 1.7, *p* = 0.002) and global longitudinal strain (17.5 ± 2.4 vs. 18.6 ± 2.2, *p* < 0.001). No significant improvement was found on re-evaluation at 1 month.

**Conclusions:**

Hospitalized patients recovering from COVID-19 were characterized by a high prevalence of left ventricular concentric remodeling, predominantly Grade I diastolic dysfunction, and a mild decrease in the longitudinal systolic function. These changes were less frequent but still prevalent in the non-hypertensive subgroup and largely persisted throughout the 1-month follow-up.

## GRAPHICAL ABSTRACT

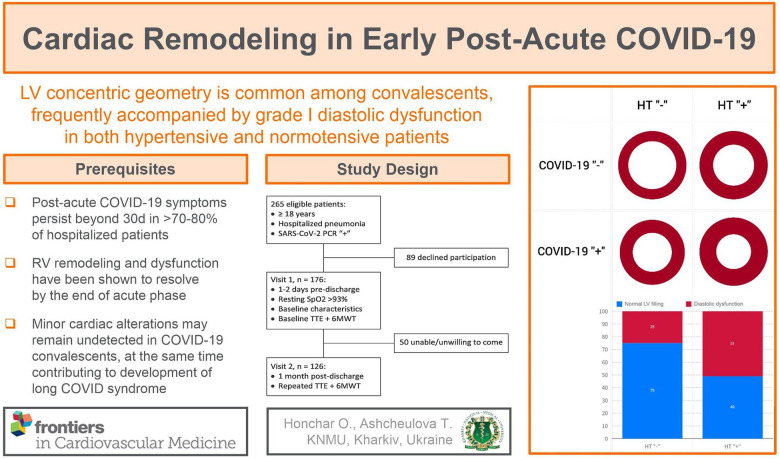

## 1. Introduction

Cardiac impairment during the acute phase of COVID-19 includes a wide spectrum of possible presentations ranging from overt cardiovascular emergencies such as acute myocardial infarction or life-threatening arrhythmias, through clearly defined clinical entities such as pulmonary embolism (PE), myo- and pericarditis, Takotsubo cardiomyopathy (1, 2), to the systemic cytokine hyperactivation mediated effects such as endothelial dysfunction, hypercoagulability, and vasoconstriction that may contribute to development of non-PE-related pulmonary hypertension and right ventricular dysfunction, microvascular ischemia resulting in left ventricular dysfunction, and form the basis for persistence of the impaired cardiac morphophysiology (3–6).

Compared to the acute phase, post-acute and chronic COVID-related cardiovascular sequelae are less thoroughly studied, and the underlying mechanisms are still not completely understood (7–9). To date, few studies using echocardiography (which is the logical first-line tool to assess cardiac structure and function) in the long COVID setting have been reported (10–19). At the same time, part of these studies were characterized by the lack of control and/or non-comprehensive echocardiographic assessment, and the emerging general picture remains at times contradictory (7).

Some of the mentioned uncertainties could be potentially related to differences in enrolled populations. Geography, gender, age, ethnicity, locally prevailing SARS-CoV-2 variants, reserve capacity of the health care system at the time the study was recruiting participants, and available logistics for the follow-up visits all inevitably affect characteristics of the observed populations in terms of disease severity and existing comorbidities, including those that have been associated with adverse prognosis both short-term in the acute COVID-19 setting (6, 20) and long-term in the general population. For instance, the prevalence of hypertension (which is the most frequent comorbidity in COVID-19 patients that is also characterized by a fairly typical phenotype of structural and functional alterations of left cardiac chambers) ranged from 15 to 57% according to different reports based on large datasets from China and the US, which, together with a similar variation in the prevalence of obesity (12–48%) and diabetes (8–34%), could at least partially account for the observed variability in echo findings (20–22).

The purpose of the current study was to identify possible echocardiographic patterns and markers of cardiac impairment in the short-term follow-up of post-acute COVID-19 patients with an additional focus on the role of hypertension as a potential confounding factor.

## 2. Material and methods

### 2.1. Study design and population

By design, this is a combined cross-sectional and longitudinal cohort study. Between January and November 2021, eligible patients who were hospitalized at the pulmonological department of Kharkiv City Hospital #13 (which is a regional pulmonological center that has been reorganized to the specialized COVID-19 care center and was serving the area of about 2.4 million people at the period of recruiting) were invited to participate in the study. Eligibility criteria included the age of ≥18 years and the diagnosis of COVID-19 pneumonia that had been confirmed with a positive polymerase chain reaction test. Exclusion criteria included stage D chronic heart failure, acute heart failure, history of myocardial infarction, permanent atrial fibrillation, stroke within 6 months, severe uncontrolled hypertension (defined as systolic BP ≥ 180 mm Hg and/or diastolic BP ≥ 110 mm Hg), significant valvular heart disease (defined as at least moderate valvular stenosis and/or at least moderate-to-severe valvular regurgitation), active cancer or systemic autoimmune pathology, inability to provide informed consent, and persisting O2 supplementation dependence by the time of discharge.

**Figure 1:**
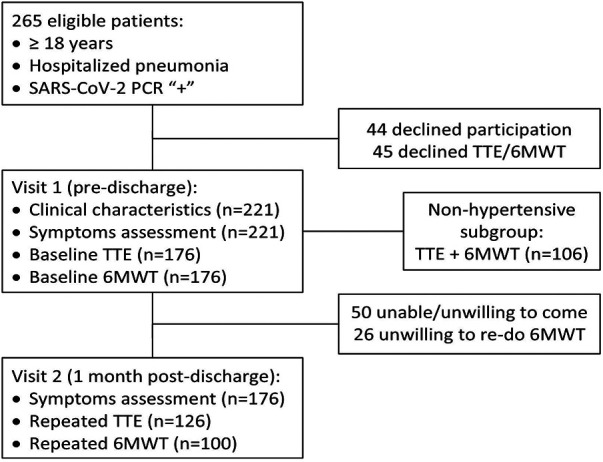
Study flowchart.

Out of a total of 265 consecutive eligible patients, 89 declined participation (mainly due to anticipated logistical difficulties in conducting the repeat visit or being reluctant to engage due to ongoing symptoms) and 176 were enrolled in the study, being a source of data for cross-sectional analysis. After the exclusion of 50 patients who were unable/unwilling to do a follow-up visit, the final cohort that was used for longitudinal comparisons included 126 participants—see Figure 1 for the study flowchart.

The control group included 88 individuals (Control 1) selected from the internal database representative of the local population from the 2018–2019 (pre-COVID) period. These subjects were individually matched to the study group at a 1:2 ratio using a nearest-neighbor strategy to adjust for age, sex, height, weight, and prevalence of hypertension and diabetes mellitus (see Table 1 for comparison on the available parameters). Considering the lack of reliable data available on the severity of hypertension and the quality of its control, a subset of self-reported non-hypertensive control subjects (Control 2, *n* = 53) was additionally compared to the cohort of non-hypertensive study participants to properly exclude the possible confounding effect of hypertension on the studied parameters.

**Table 1:** Baseline characteristics of the study population and its non-hypertensive subgroup vs. control.

Parameters	COVID-19 general, *n* = 176	Control 1, *n* = 88	*P*-values	COVID-19 non-HT, *n* = 106	Control 2, *n* = 53	*P*-values
Age	53.4 ± 13.6	52.3 ± 13.3	0.51	50.7 ± 13.9	53.0 ± 14.5	0.32
Female sex	93 (52.8)	46 (52.3)	0.97	55 (51.9)	28 (52.3)	0.91
Height, cm	169.8 ± 9.1	170.6 ± 7.6	0.47	169.3 ± 8.7	170.0 ± 6.9	0.64
Weight, kg	84.5 ± 18.5	85.6 ± 16.7	0.64	78.9 ± 15.8	79.0 ± 11.9	0.98
BMI, kg/m^2^	29.1 ± 5.2	29.3 ± 4.9	0.76	27.4 ± 4.4	27.3 ± 3.5	0.85
Hypertension	70 (39.8)	34 (38.6)	0.96	0 (0.0)	0 (0.0)	1.00
Obesity	67 (38.1)	35 (39.8)	0.89	25 (23.6)	13 (24.5)	0.90
Diabetes	17 (9.6)	8 (9.1)	0.94	2 (1.9)	1 (1.9)	1.00

BMI, body mass index. Matching quality was preserved for the cohort of patients who underwent repeated evaluation (*n* = 126) with *P*-values ≥ 0.83 for all used parameters.

### 2.2. Clinical data collection

The first visit was performed 1–2 days before discharge, after stabilization of patients’ clinical condition (capillary blood oxygen saturation >93% on room air) and achievement of clinical criteria of epidemic safety (normal body temperature and absence of acute respiratory disease symptoms for ≥3 days starting from the 10th day after onset of symptoms) (23). During this visit, demographic characteristics (age, gender), data on laboratory parameters, computed tomography findings, and treatment were obtained from the medical records, data on symptoms, smoking status, and comorbidities were collected by interview, and anthropometry was performed, followed by comprehensive transthoracic echocardiography (TTE). 6 min walk distance (6MWD) was assessed using a 20 m track; respective log-linear models (24) were used to calculate the individual predicted values.

The follow-up visit for re-assessment of symptoms, changes in clinical parameters, structural and functional state of the cardiovascular system was carried out at 1 month.

### 2.3. Echocardiography

Transthoracic echocardiography was performed using the Radmir ULTIMA Expert ultrasound system (Radmir Co., Ukraine). Linear and volumetric measurements were performed in accordance with the current guidelines for chamber quantification by the American Society of Echocardiography (ASE) and the European Association of Cardiovascular Imaging (EACVI) (25). Linear left ventricular (LV) dimensions and walls’ thickness were obtained using 2D measurements in the parasternal long-axis view, and LV end-diastolic length (LV L) in the apical 4-chamber view. Left ventricular end-diastolic (EDV) and end-systolic (ESV) volumes and ejection fraction (EF), as well as left atrial volume, were measured in the apical 4- and 2-chamber views using Simpson’s biplane method. Tricuspid (TAPSE) and mitral (MAPSE) annular plane systolic excursion were measured using M-mode in the apical 4-chamber view, with MAPSE being calculated as a mean value of excursion of its lateral and medial portions.

Left ventricular global longitudinal strain was calculated as LV GLS = MAPSE/LVL* 100% using the recently proposed linear method (26–28). Similarly, right ventricular free wall longitudinal strain was calculated as RVLS = TAPSE/RVL* 100%. LV GLS and RVLS are reported as absolute values.

Mitral and tricuspid annular motion velocities, as well as parameters of transmitral and transtricuspid blood flow, were measured in pulsed-wave tissue Doppler mode according to the standard methods provided in the current guidelines (29, 30).

Diagnosis and grading of the left ventricular diastolic dysfunction (DD) were performed in accordance with the 2016 ASE/EACVI algorithm (29) with inclusion of the myocardial disease concept; in cases of lacking data on tricuspid regurgitation velocity, we only ruled patients as having DD when present structural abnormality was corroborated by tissue Doppler findings (i.e., isolated left atrial dilation or LV remodeling were not considered signs of DD).

### 2.4. Statistical analysis

The collected data was analyzed using StatSoft STATISTICA Version 12 statistical analysis software package. Data distribution was assessed using Shapiro-Wilk test. For all variables, descriptive statistics are reported as mean ± standard deviation (SD) or median [interquartile range] for normally distributed and skewed continuous variables, respectively. Categorical variables are reported as counts (percentages). Cross-sectional comparisons of continuous variables were performed using independent samples *t*-test for normally distributed parameters and Mann-Whitney *U*-test for skewed variables; Chi-Square test was used to compare binary and categorical variables. Longitudinal comparisons were made using paired samples *t*-test or Wilcoxon signed-rank test. *P* values reported were calculated using two-sided Fisher’s exact test, the differences were considered significant if *P* < 0.05. Correlation analysis was performed using the linear Pearson method.

## 3. Results

### 3.1. Clinical characteristics

The mean age of the initial 176 participants who entered the study (including 53% female and 47% male patients) was 53.4 ± 13.6 years. The average time from symptoms onset was 22.6 ± 7.1 days for Visit 1 and 54.3 ± 8.2 days for Visit 2. The most frequent comorbidities were hypertension and obesity with the prevalence of both close to 40%; the complete report on comorbidities and further clinical characteristics are presented in Table 2.

**Table 2:** Clinical characteristics of pre-discharge COVID-19 patients who participated in the study in comparison with matched control.

Active smoking status pre-disease, pack years	29 (16.5) 1 [1; 15]
Comorbidities	
Hypertension	70 (39.8)
Obesity	67 (38.1)
Diabetes mellitus, type 2	17 (9.6)
Chronic obstructive pulmonary disease	5 (2.8)
Bronchial asthma	4 (2.3)
Pulmonary emphysema	3 (1.7)
Angina pectoris	3 (1.7)
History of stroke/TIA	6 (3.4)
Chronic kidney disease	5 (2.8)
Chronic liver disease	2 (1.1)
History of peptic ulcer	13 (7.4)
History of cancer	10 (5.7)
Charlson comorbidity index	0.5 ± 0.8
Minimal SpO_2_, %	89 [85; 94]
Pulmonary tissue involvement by CT*, %	32.5 ± 20.2
Laboratory parameters	
Peak IL-6, pg/ml	10.0 [3,1; 25.2]
Peak CRP, mg/L	24.0 [7,3; 55.0]
Peak ESR, mm/h	30 [20; 40]
Peak procalcitonin, ng/ml	0.06 [0,04; 0.12]
Peak D-dimer, ng/ml	278 [154; 508]
Oxygen supplementation	
Via nasal cannula	101 (57.4)
Noninvasive/invasive ventilation	9 (5.1)
Treatment	
Methylprednisolone pulse therapy	115 (65.3)
Dexamethasone	155 (88.1)
Remdesivir	82 (46.6)

* Assessment was performed using the methodology for the simplified RALE score as proposed by Wong et al. (44), mean value of the reported % range was taken for analysis; BMI, body mass index; TIA, transient ischemic attack; SpO_2_, peripheral capillary oxygen saturation; IL-6, interleukin 6; CRP, C-reactive protein; ESR, erythrocyte sedimentation rate.

### 3.2. Echocardiographic data at baseline

Echocardiographic assessment of cardiac structure in observed patients has revealed a mild increase in LA size and volume, interventricular septum (IVS) and posterior LV wall thickness, and myocardial mass parameters compared to matched control (see Table 3). The observed changes resulted in a high proportion of patients with concentric LV geometry, whereas the LV hypertrophy rate was insignificantly increased vs. control and remained generally in line with common knowledge of its prevalence in the European population (31).

**Table 3:** Echocardiographic characteristic of the study participants.

Parameters	COVID-19 general, *n* = 176	Control 1, *n* = 88	Difference (95% CI)	2-sided *p*
Left chambers morphometry				
LA size, mm	37.6 ± 4.0	36.4 ± 3.5	1.3 (0.3; 2.3)	0.010
LA volume index, ml/m^2^	28.6 ± 6.6	25.1 ± 4.9	3.5 (1.9; 5.1)	<0.001
Interventricular septum, mm	10.3 ± 1.6	9.1 ± 1.0	1.3 (0.9; 1.6)	<0.001
LV posterior wall, mm	9.9 ± 1.3	9.0 ± 0.8	0.9 (0.6; 1.2)	<0.001
LV relative wall thickness	0.45 ± 0.07	0.39 ± 0.04	0.07 (0.05; 0.08)	<0.001
LV end-diastolic diameter, mm	45.2 ± 4.0	46.9 ± 3.3	−1.8 (−3.1; −0.4)	<0.001
LV end-systolic diameter, mm	28.9 ± 3.5	31.1 ± 2.5	−2.2 (−3.0; −1.4)	<0.001
LV length, mm	81.1 ± 7.2	81.7 ± 6.2	−0.6 (−2.2; 1.0)	0.505
LV mass index (BSA), g/m^2^	81.4 ± 16.9	72.8 ± 10.2	8.6 (4.7; 12.4)	<0.001
LV mass index (height^2,7^), g/m^2,7^	38.1 ± 8.9	33.9 ± 5.8	4.1 (2.1; 6.2)	<0.001
LV concentric geometry	104 (59.1)	15 (17.0)		<0.001
LV hypertrophy	21 (11.9)	6 (6.8)		0.196
Left ventricular systolic function				
LV ejection fraction, %	65.3 ± 6.7	62.2 ± 4.6	3.2 (1.0; 5.4)	<0.001
MAPSE, mm	14.2 ± 2.3	15.1 ± 2.1	−1.0 (−1.5; −0.3)	0.002
LV global longitudinal strain, %	17.5 ± 2.4	18.6 ± 2.2	−1.0 (−1.6; −0.4)	<0.001
LV midwall shortening, %	15.7 ± 2.1	16.4 ± 1.9	−0.7 (−1.2; −0.2)	0.006
LV stroke volume index, ml/m^2^	31.7 ± 6.4	32.3 ± 5.7	−0.6 (−2.1; −1.0)	0.495
LV s’, cm/s	9.7 ± 1.7	10.0 ± 1.4	−0.3 (−0.7; 0.1)	0.199
Left ventricular diastolic function				
LV e’, cm/s	9.2 ± 2.2	11.3 ± 2.6	−2.1 (−2.7; −1.5)	<0.001
LV E, cm/s	67.4 ± 17.2	74.3 ± 16.0	−6.9 (−11.3; −2.6)	0.002
LV E/A ratio	1.01 ± 0.26	1.09 ± 0.33	−0.07 (−0.15; 0.00)	0.055
LV E/e’ ratio	7.5 ± 1.8	6.8 ± 1.7	0.7 (0.3; 1.2)	0.002
LV diastolic dysfunction	62 (35.2)	12 (13.6)		<0.001
Right chambers evaluation				
RA size, mm	35.8 ± 3.8	36.6 ± 4.1	−0.8 (−1.8; 0.2)	0.110
RV size (proximal outflow tract)	31.8 ± 3.3	32.4 ± 3.5	−0.6 (−1.6; 0.3)	0.164
TAPSE, mm	24.8 ± 4.3	25.6 ± 3.9	−0.8 (−1.9; 0.3)	0.143

CI, confidence interval; LA, left atrium; LV, left ventricle; BSA, body surface area; MAPSE, mitral annular plane systolic excursion; RA, right atrium; RV, right ventricle; TAPSE, tricuspid annular plane systolic excursion.

Assessment of the LV systolic parameters in the study cohort has revealed a mild decrease in the B-mode derived indices of longitudinal function (MAPSE and GLS) and the midwall shortening vs. control. At the same time, a minimal increase in ejection fraction in the setting of a mildly decreased LV cavity resulted in the absence of changes in the cardiac output as assessed by the stroke volume index.

LV diastolic function was characterized by a 20% reduction of mean e’ velocities, reaching subnormal values in 47% of participants. An E/e’ increase that was observed, however, was of little magnitude, leaving the vast majority of study subjects well below the cut-off values suggestive of increased LV filling pressures. The lack of traceable tricuspid regurgitation in the majority of patients (together with no evidence of right chambers remodeling or dysfunction) suggested normal pulmonary artery pressures but complicated the grading of diastolic dysfunction. As a result, out of 62 patients with diastolic dysfunction, 47 (26.7%) were categorized as Grade I DD and 15 (8.5%) as having indeterminate filling pressures. In the absence of data on left atrial strain, re-classification of these patients using LV GLS values (29, 32) with a cut-off of 16% (33) has allowed us to identify 7 (4.0%) subjects with apparently increased filling pressures.

Considering the similarities of changes in cardiac morphology and function that we observed in the study group to the hypertensive phenotype, a sub-analysis focused on the cohort of non-hypertensive participants (*n* = 106) was additionally performed to completely eliminate the possible confounding effect of differences in hypertension severity and quality of its control.

In the proposed setting, we observed less pronounced changes that were still similar to the concentric phenotype described above (see Table 4). Despite the 0.5 mm lesser absolute LV wall thickness, their mean values were still higher vs. control, as was the relative wall thickness, resulting in a 43% prevalence of concentric LV geometry. Myocardial mass parameters were also mildly increased, showing intermediate values between hypertensive participants (85.6 ± 21.0 g/m^2^ for BSA-indexed LV myocardial mass in the latter) and the control group.

**Table 4:** Echocardiographic characteristic of non-hypertensive hospitalized COVID−19 patients.

Parameters	COVID-19 non-HT, *n* = 106	Control 2, *n* = 53	Difference (95% CI)	2-sided *p*
Left chambers morphometry				
LA size, mm	36.3 ± 3.7	35.2 ± 3.1	1.1 (0.1; 2.3)	0.064
LA volume index, ml/m^2^	28.5 ± 6.8	25.4 ± 5.5	3.1 (1.0; 5.2)	0.004
Interventricular septum, mm	9.7 ± 1.2	8.8 ± 0.9	0.9 (0.5; 1.3)	<0.001
LV posterior wall, mm	9.4 ± 1.1	8.8 ± 0.8	0.6 (0.3; 1.0)	<0.001
LV relative wall thickness	0.42 ± 0.05	0.38 ± 0.04	0.04 (0.02; 0.06)	<0.001
LV end-diastolic diameter, mm	45.5 ± 3.5	46.3 ± 3.1	−0.8 (−1.9; 0.4)	0.178
LV end-systolic diameter, mm	29.4 ± 3.7	30.9 ± 2.6	−1.5 (−2.6; −0.4)	0.009
LV length, mm	80.3 ± 6.1	81.0 ± 5.5	−0.8 (−2.5; 9.7)	0.365
LV mass index (BSA), g/m^2^	78.4 ± 14.0	71.5 ± 10.2	6.9 (2.6; 11.2)	0.002
LV mass index (height^2,7^), g/m^2,7^	35.6 ± 6.8	32.4 ± 4.9	3.4 (1.3; 5.4)	0.002
LV concentric geometry	46 (43.4)	7 (13.2)		<0.001
LV hypertrophy	4 (3.8)	0 (0)		0.371
Left ventricular systolic function				
LV ejection fraction, %	64.7 ± 7.1	61.9 ± 4.7	2.9 (0.8; 5.0)	0.008
MAPSE, mm	14.2 ± 2.1	15.0 ± 2.0	−0.8 (−1.4; −0.2)	0.023
LV global longitudinal strain, %	17.8 ± 2.3	18.5 ± 2.2	−0.7 (−1.4; −0.2)	0.068*
LV midwall shortening, %	16.2 ± 1.8	16.5 ± 1.8	−0.3 (−0.9; 0.3)	0.310
LV stroke volume index, ml/m^2^	32.8 ± 5.9	32.4 ± 5.6	0.3 (−1.6; 2.2)	0.755
LV s’, cm/s	9.7 ± 1.7	9.9 ± 1.4	−0.2 (−0.7; 0.3)	0.465
Left ventricular diastolic function				
LV e’, cm/s	9.8 ± 2.2	11.5 ± 2.5	−1.8 (−2.5; −1.0)	<0.001
LV E, cm/s	68.6 ± 18.9	71.6 ± 14.4	−6.9 (−11.3; −2.6)	0.311
LV E/A ratio	1.09 ± 0.28	1.09 ± 0.32	−0.07 (−0.15; 0.00)	0.876
LV E/e’ ratio	7.1 ± 1.6	6.4 ± 1.5	0.7 (0.2; 1.3)	0.006
LV diastolic dysfunction	27 (25.5)	5 (9.4)		0.017
Right chambers evaluation				
RA size, mm	35.9 ± 3.7	36.1 ± 4.4	−0.2 (−1.5; 1.1)	0.308
RV size (proximal outflow tract)	30.8 ± 2.9	31.9 ± 3.6	−1.1 (−2.3; 0.0)	0.048
TAPSE, mm	24.7 ± 4.1	25.4 ± 3.9	−0.8 (−1.9; 0.3)	0.304

* 1-sided *p* = 0,034. CI, confidence interval; LA, left atrium; LV, left ventricle; BSA, body surface area; MAPSE, mitral annular plane systolic excursion; RA, right atrium; RV, right ventricle; TAPSE, tricuspid annular plane systolic excursion.

A similar pattern was observed when assessing LV diastolic filling, with a statistically significant decrease in mitral e’ velocity and an increase in E/e’ ratio resulting in a 25% prevalence of Grade I diastolic dysfunction. MAPSE and GLS values were also mildly decreased in non-hypertensive COVID-19 patients on the background of a clinically insignificant increase in ejection fraction.

### 3.3. 1-Month follow-up

Another aspect of our study was focused on the assessment of short-term post-discharge dynamics of echocardiographic parameters in observed COVID-19 patients based on the results of repeated comprehensive transthoracic echocardiography after a median of 31 days from the first visit. Table 5 summarizes the obtained results and presents data on the comparison of Visit 2 parameters vs. control.

**Table 5:** Results of a 1-month echocardiographic follow-up of observed patients with COVID-19.

Parameters	COVID-19 general		Visit 2 vs. Visit 1		Visit 2 vs. Control 1	
	Visit 1	Visit 2	Difference (95% CI)	2-sided *p*	Difference (95% CI)	2-sided *p*
Left chambers morphometry						
LA size, mm	37.5 ± 4.0	37.5 ± 4.6	0.0 (−0.5; 0.5)	0.920	1.1 (0.0; 2.3)	0.053
LA volume index, ml/m^2^	28.3 ± 5.8	27.5 ± 6.2	−0.8 (−1.9; 0.3)	0.154	2.3 (1,0; 3,7)	<0.001
Interventricular septum, mm	10.3 ± 1.5	10.1 ± 1.4	−0.2 (−0.3; 0.0)	0.031	1.0 (0.7; 1.4)	<0.001
LV posterior wall, mm	9.9 ± 1.3	9.9 ± 1.3	0.0 (−0.2; 0.1)	0.569	0.9 (0.5; 1.2)	<0.001
LV relative wall thickness	0.45 ± 0.08	0.45 ± 0.08	0.00 (−0.01; 0.01)	0.340	0.06 (0.05; 0.08)	<0.001
LV end-diastolic diameter, mm	44.8 ± 4.0	44.8 ± 4.2	0.0 (−0.4; 0.4)	0.965	−2.0 (−3.1; −1.0)	<0.001
LV end-systolic diameter, mm	28.6 ± 3.6	29.0 ± 4.0	0.4 (0.0; 0.8)	0.034	−2.2 (−3.1; −1.2)	<0.001
LV mass, g	155.6 ± 35.4	153.3 ± 34.2	−2.3 (−5.1; 0.5)	0.102	9.2 (0.4; 17.9)	0.040
LV mass index (BSA), g/m^2^	79.5 ± 14.2	77.3 ± 12.2	−2.2 (−3.6; 0.8)	0.002	4.5 (1.3; 7.6)	0.005
LV mass index (H^2,7^), g/m^2,7^	36.8 ± 7.7	36.2 ± 7.1	−0.6 (−1.2; 0.1)	0.085	2.3 (0.5; 4.1)	0.013
LV concentric geometry	74 (58.7)	81 (64.3)		0.437		<0.001
LV hypertrophy	11 (8.7)	11 (8.7)		1.000		0.801
Left ventricular systolic function						
LV ejection fraction, %	65.7 ± 6.8	64.6 ± 6.8	−1.0 (1.0; 5.4)	<0.001	2.7 (1.0; 4.3)	0.002
MAPSE, mm	14.1 ± 2.1	14.1 ± 2.2	0.0 (−0.5; 0.4)	0.823	−1.1 (−1.6; −0.6)	<0.001
LV GLS, %	17.3 ± 2.4	17.3 ± 2.1	0.0 (−0.4; 0.4)	0.879	−1.3 (−1.9; −0.7)	<0.001
LV midwall shortening, %	15.7 ± 2.1	15.5 ± 2.0	−0.2 (−1.2; −0.2)	0.006	−0.9 (−1.4; −0.4)	0.001
LV SVI, ml/m^2^	31.2 ± 6.3	30.1 ± 5.3	−1.0 (−2.1; 0.0)	0.060	−2.2 (−3.7; −0.6)	0.005
Minute volume of blood, L	5.15 ± 1.54	4.89 ± 1.21	−0.26 (−0.49; 0.02)	0.031		
LV s’, cm/s	9.7 ± 1.9	9.3 ± 1.7	−0.4 (−0.7; 0.2)	0.001	−0.7 (−1.2; −0.3)	0.001
Left ventricular diastolic function						
LV e’, cm/s	9.5 ± 2.3	9.8 ± 2.8	0.2 (−0.1; 0.6)	0.131	−1.5 (−2.3; −0.8)	<0.001
LV E, cm/s	69.6 ± 17.6	67.9 ± 15.7	−1.7 (−4.8; 1.4)	0.271	−6.5 (−10.8; −2.1)	0.004
LV E/A ratio	1.06 ± 0.26	1.03 ± 0.29	−0.02 (−0.07; 0.02)	0.328	−0.05 (−0.14; 0.03)	0.224
LV E/e’ ratio	7.5 ± 1.7	7.3 ± 2.3	−0.1 (−0.4; 0.1)	0.300	0.6 (0.0; 1.1)	0.055
LV diastolic dysfunction	39 (31.0)	46 (36.5)		0.424		<0.001
Right chambers evaluation						
RA size, mm	36.1 ± 4.1	35.7 ± 4.1	−0.3 (−1.1; 0.4)	0.365	−0.9 (−2.0; 0.3)	0.130
RA area index, mm^2^/m^2^	8.3 ± 2.2	7.7 ± 1.4	−0.6 (−1.0; 0.1)	0.014		
RV size	31.8 ± 3.3	31.9 ± 3.3	0.1 (−0.4; 0.6)	0.751	−0.5 (−1.5; 0.6)	0.378
TAPSE, mm	24.8 ± 4.1	24.0 ± 3.5	−0.8 (−1.5; 0.1)	0.020	−1.4 (−2.5; −0.4)	0.006
RVLS, %	36.2 ± 6.4	34.8 ± 5.9	−1.4 (−2.5; 0.3)	0.010		
RV s’, cm/s	14.6 ± 2.6	14.0 ± 2.6	−0.7 (−1.2; 0.1)	0.014		
RV e’, cm/s	11.3 ± 2.3	10.9 ± 2.1	−0.4 (−0.9; 0.1)	0.086		
RV E/e’ ratio	4.4 ± 1.3	4.5 ± 1.1	0.1 (−0.1; 0.4)	0.236		

CI, confidence interval; LA, left atrium; LV, left ventricle; BSA, body surface area; MAPSE, mitral annular plane systolic excursion; GLS, global longitudinal strain; SVI, stroke volume index; RA, right atrium; RV, right ventricle; TAPSE, tricuspid annular plane systolic excursion; RVLS, RV free wall longitudinal strain.

Despite the natural post-hospitalization reconditioning resulting in a previously reported increase in the 6MWD among the study participants from 401 ± 71 to 463 ± 65 m (62.7 ± 10.6–74.0 ± 11.1% of the predicted values, *p* < 0,001 for both indices) during a one-month follow-up (34), we were only able to detect minimal dynamic changes in cardiac morphology. Those were limited to a 2% decrease in the interventricular septum thickness which resulted in a borderline decrease in myocardial mass parameters compared to Visit 1. Evaluation of cardiac function revealed a minimal decrease in the estimated minute volume of blood that was associated with unidirectional and proportional (circa −3 to −5%) change of most systolic parameters vs. baseline, including LV ejection fraction and midwall shortening, RV free wall longitudinal strain, TAPSE, and both mitral and tricuspid annular s’ velocities, accompanied with a minimal increase in LV end-systolic diameter. Assessment of the diastolic filling of both ventricles did not reveal any significant changes during a short-term follow-up.

Thus, the observed cohort of COVID-19 patients at the time point of 1 month after discharge has retained the features indicative of the shift towards concentric LV geometry (an increase in absolute and relative wall thickness and higher values of myocardial mass indices), with RWT reaching values >0,42 in 64% of participants, including 55% of non-hypertensive subjects. These changes were accompanied by a mild depression of ventricular longitudinal function, manifested as a persisting 5%–10% decrease in LV GLS, MAPSE, TAPSE, and mitral annular velocities vs. control. LV diastolic dysfunction remained highly prevalent and was detected in 36% of cases in the general cohort and 30%—among non-hypertensive participants.

Out of the parameters assessed in our study, it was the LV wall absolute and relative thickness and myocardial mass parameters at Visit 1 that had a weak-to-moderate, but significant correlation with the increase in the reached percent of predicted 6-minute walk distance during the follow-up period—the strongest links were detected for interventricular septum (*r* = 0.37) and LV MMI by height^2,7^ (*r* = 0.31). These findings implied that those were the patients with initially thicker walls who could potentially gain higher 6MWD increment vs. those in whom LV walls had a closer to normal thickness by the moment of discharge. Correlation analysis of dynamic changes in LV morphology has confirmed these suggestions, showing that the only parameters related to the increase in the 6 min walk distance % were a decrease in the IVS thickness (*r* = −0.31) and LV myocardial mass / MMI by height^2,7^ (*r* = −0.31). (See Supplementary Table S1 for the detailed report on revealed correlations).

## 4. Discussion

A lot of attention has been drawn recently to the problem of long COVID syndrome (35–38). Compared to the acute phase, underlying pathogenetic mechanisms are less profoundly known, with limited and at times conflicting data available on the specific features of post-acute cardiovascular sequelae of COVID-19 (7–9).

This combined cross-sectional case-control and longitudinal cohort study reports the results of comprehensive transthoracic echocardiographic assessment in hospitalized COVID-19 patients that was performed 1–2 days pre-discharge at the baseline and repeated after a 1-month follow-up.

The main findings included a high prevalence of concentric LV geometry that was present in 59% of participants, including 43% prevalence in the non-hypertensive subgroup, and predominantly Grade I diastolic dysfunction that was found in 35% and 25% of patients, respectively, presenting a significant difference compared to the age-, sex-, height-, weight-, and comorbidities-matched control. The observed changes persisted throughout the follow-up period, showing no significant improvement at 1 month.

Other findings naturally followed from the described LV phenotype and included a mild relative increase in LV wall thickness and myocardial mass parameters vs. control and a mild decrease in the indices of both diastolic and longitudinal systolic ventricular function. The magnitude of these changes, however, was little, leaving the mean values (except wall thickness) within normal limits and therefore being hardly clinically significant when taken isolated.

In the assessment of the biventricular longitudinal systolic function, we applied a recently proposed linear method that was later validated on 1266 cardiovascular disease-free individuals in the HUNT study, showing a close to linear correlation and no significant differences to the 2D speckle tracking-obtained values (26–28). The advantages of the selected method include universal availability, vendor independence, and low dependence on the image quality, which allowed us to obtain valid results in 100% of participants.

Most of the echocardiographic studies that had been performed to date in COVID-19 patients were focused on the assessment of changes in cardiac structure and function during the acute phase of disease and on the evaluation of their prognostic significance, mostly using a hard endpoint of COVID-19-related death (14, 39–41). The main findings on focused TTE that was usually used in this setting included RV dilation and dysfunction in a significant proportion of the patients, followed by LV functional alterations that in case of being clinically significant were typically related to pre-existing cardiac pathology. RV dilation and strain values, TAPSE, and LV GLS were most frequently identified as independent predictors of mortality. At the same time, it is worth noting in the context of our study that the LV diastolic dimensions, when reported, were typically less compared to the usual values in the general population, with mean values reaching as low as 42–43 mm when performed early during the hospitalization period (39, 41) and coming closer to 45 mm when examining patients later (14, 40). Szekely et al. also report in detail on LV tissue Doppler parameters obtained during 24 h from hospitalization that included low mean values of mitral annular velocities (7.4 cm/s for both s’ and e’) and a high E/e’ ratio (weighted mean 10.4 for all patients), most likely indicating a high prevalence of diastolic dysfunction (41).

The study by Moody et al. (14) is among the few that included a baseline in-hospital echocardiographic evaluation (at a median of 8 days after admission) with subsequent longitudinal follow-up; similar to most of the above, it also used a focused TTE protocol and therefore did not report on most of the parameters that were evaluated in our study. However, the results reported on the LV end-diastolic diameter were similar to those observed by us, and a high prevalence of RV overload and dysfunction (defined as TAPSE <17 mm) that was not detected in our study was mostly resolved by the moment of re-evaluation at 3 months.

Out of studies available on recovery after COVID-19, few have used comprehensive sonographic assessment of cardiac structure and function. In a study by Catena et al. (11), the authors report LV morphology features in patients who were troponin-negative at hospitalization that are virtually indistinguishable from those that we observed in the control group. Even more interesting is the fact that troponin-positive patients in that study displayed a clear tendency to an increase in the LV wall thickness and myocardial mass index, despite the differences not reaching the significance levels (most likely due to the low number of patients, *n* = 18). Similar findings were observed for mitral annular e’ velocity and E/e’ ratio that were equal between troponin-negative patients and Control 1 subjects in our study but insignificantly deteriorated in ex-troponin-positive participants in (11). The values of LV ejection fraction, being somewhat higher compared to our study, were also characterized by a minor increase in a “worse” clinical group. At the same time, the authors report no difference in MAPSE and TAPSE, presenting values that are lower compared to both groups in our study.

In another development by the same team, Sechi et al. compare the same general cohort of 105 hospitalized COVID-19 patients to a set of 1:1 matched control subjects, providing to date the most detailed echocardiographic characterization of the latter that was obtained in the closest setting to that of our study (at a median of 41 days from the date of COVID-19 diagnosis) (12). An indirect comparison of our results to the presented data has both corroborated the findings in the main group and allowed us to externally validate the control group used in our study—it was characterized by a minimal uni-directed shift towards “more concentric” geometry vs. controls in (12), thus being unlikely to partially account for the observed differences vs. study group (see Supplementary Table S2). Similar to our results, the authors demonstrate (an insignificant) tendency to increase in the LV relative wall thickness due to a minimal decrease in end-diastolic diameter. Contrary to our findings, no significant changes have been reported regarding the longitudinal ventricular function despite the almost identical values of MAPSE both in the main and control groups to those in our study.

Another study by Ingul et al. (16) presents a comprehensive functional echocardiographic assessment of 204 COVID-19 survivors that was performed at 3 months follow-up using the comparison to the age-, sex-, BMI-, systolic blood pressure- and comorbidities-matched control. Similar to our findings, it reports a decrease in LV end-diastolic volume index, which should have been accompanied by increased relative wall thickness unless associated with proportionally lower myocardial mass. Other corroborating results include the evidence of a relatively poorer biventricular longitudinal function, manifested as a mild but statistically significant decrease in MAPSE, mitral e’ velocity, TAPSE, and RV free wall strain; despite a minimal decrease in ejection fraction, LV GLS is reported to be paradoxically higher vs. control, being interpreted by authors as a clinically insignificant sign that could be operator dependent.

Tudoran et al. (19) report a high prevalence of LV diastolic dysfunction among patients with long COVID syndrome, reaching 63% in obese and 22% in non-obese subjects. Despite the important discrepancies in the study population (selected long COVID vs. general COVID-19 cohort; mainly outpatient management vs. hospitalized patients), participants were enrolled at 4–10 weeks from the onset of symptoms, which was approximating the timing of Visit 2 in our study, and adjustment of the mentioned rates to the prevalence of obesity in our cohort results in a weighted mean rate of diastolic dysfunction of 38%, coming very close to the factual prevalence that we observed.

We did not identify prior studies longitudinally assessing echocardiographic dynamics within the early post-acute period of COVID-19 that would not use the parameters obtained during the early acute phase as a baseline. The current study presents data on the 1-month follow-up in the period from a median of 23–54 days after manifestation of symptoms, demonstrating no significant changes in cardiac morphology during this phase. The minimal decrease in the systolic indices that we observed was not associated with clinical deterioration and most likely resulted from the gradual reversal of a somewhat higher output state due to SARS-CoV-2-induced pulmonary affection. The same process could have explained the tendency to a minimal decrease in myocardial mass parameters (the observed changes of BSA-indexed LV MMI were partially driven by the restoration of the weight loss during the acute phase—in the follow-up period the patients gained 2.6 ± 3.3 kg). At the same time, the observed minimal changes in myocardial mass were contrasted with the expected dynamics during the period of post-hospitalization reconditioning (42) and could also be hypothetically explained (along with an increase in myocardial mass vs. control) by mild myocardial edema persisting in some patients [as shown in series of cardiac magnetic resonance studies (43)]. In this scenario, its gradual resolution during the observation period could potentially explain the correlations found in our study between the decrease in IVS thickness/myocardial mass and the improvement in 6MWD.

In the report on a long-term echocardiographic follow-up at a later phase after hospitalization for COVID-19, Ovrebotten et al. (17) demonstrated no changes in both LV and RV parameters (including morphometry and longitudinal strain) during the period from 3 to 12 months. These findings, along with the results of our study, suggest that identification of patients with delayed/incomplete resolution of minor COVID-19-related cardiac alterations may be performed as early as during the late acute/early post-acute period of SARS-CoV-2 infection.

### 4.1. Strengths and limitations

To our knowledge, this is the first study focusing on the comprehensive assessment of echocardiographic changes in hospitalized COVID-19 patients during the early post-discharge period. Simultaneous cross-sectional comparison to the matched control allowed us to more clearly outline the existing minor changes in the cardiac structure and function that still appeared to be linked to the observed functional improvement during one month of follow-up. A sub-analysis that was performed in a cohort of non-hypertensive individuals has allowed us to more reliably mitigate the confounding effect of hypertension compared to the simple matching of its self-reported presence, given that the latter does not account for the possible differences in the severity and control of hypertension.

Being a single-center study, it could be susceptible to hospital-related confounding effects. The most notable difference between the used treatment and the commonly applied standards was the high proportion of patients who received pulse therapy with Methylprednisolone during the first days of hospitalization. However, one would expect its possible effects on cardiac geometry and function to be transitory if existing at all, and to resolve by the moment of baseline evaluation, which was confirmed by the absence of any correlations between its use and the studied parameters. There was a source of selection bias in the design and protocol of the study—candidates with severe underlying cardiac pathology were specifically excluded for possible subtle changes in evaluated parameters not to get confounded by more severe manifestations of pre-existing disease that would be hard to adequately match to control. In addition, patients with a more severe course of COVID-19 could have been less likely to participate due to being still oxygen-dependent by the moment of discharge or reluctant to leave the floor and/or to engage in the 6 min walk test, and thus the study population might not truly reflect the characteristics of the general mass of consecutively hospitalized patients. Lastly, the prevailing SARS-CoV-2 variants at the time of enrollment were different compared to more recent time, and a higher proportion of patients were not vaccinated, mandating caution in generalizing the obtained results to the current setting of post-acute COVID-19 care.

## 5. Conclusions

Hospitalized patients recovering from COVID-19 were characterized by the high prevalence of LV concentric remodeling, predominantly Grade I diastolic dysfunction, and a mild decrease in the longitudinal systolic function compared to matched control. The changes in LV geometry and diastolic dysfunction were less frequent but still prevalent in the non-hypertensive subgroup. The observed changes largely persisted during a one-month follow-up showing no general tendency to improvement, with a minor decrease in the IVS thickness and LV mass index correlating with an increase in the 6MWD.

## Data Availability

The raw data supporting the conclusions of this article will be made available by the authors, without undue reservation.
