# Supplementary material for: Short-term echocardiographic follow-up after hospitalization for COVID-19: a focus on early post-acute changes
Source: Front Cardiovasc Med. 2023 Nov 22;10:1250656. doi: 10.3389/fcvm.2023.1250656 (PMC10703357; doi:10.3389/fcvm.2023.1250656)
Supplement: Supplementary file 1 [file Datasheet1.pdf]

Supplementary Material

Short-Term Echocardiographic Follow-Up After Hospitalization for COVID-19: A Focus on Early Post-Acute Changes

Oleksii Honchar*, **Tetiana Ashcheulova**

*** Correspondence:**
Corresponding Author
ov.honchar@knmu.edu.ua

Supplementary Tables

Supplementary table 1. Correlations between echocardiographic parameters and 6-minute walk distance at both visits in the general study group.

Variable	16MWD	16MWD%	26MWD	26MWD%	D.6MWD	D.6MWD%
1LA	-0,085258	0,055178	-0,129919	0,102634	0,016850	0,084326
1LAVI	-0,135352	0,223344	-0,251309	0,262419	-0,043472	0,050416
1IVS	-0,340884	-0,091883	-0,342676	0,050467	0,199327	0,375116
1LVPW	-0,342267	-0,080858	-0,382569	0,021996	0,233114	0,315089
1LV RWT	-0,383911	-0,037889	-0,427800	0,074970	0,119524	0,224786
1LV EDD	0,134834	-0,094945	0,212082	-0,123799	0,074126	-0,009273
1LV ESD	0,309595	0,012158	0,293044	-0,140290	-0,021785	-0,108398
1MAPSEmed	0,061124	-0,139538	0,230430	0,134890	0,319252	0,280919
1MAPSElat	0,034289	-0,106563	0,132418	0,028215	0,271386	0,206026
1LV GLS	-0,074629	-0,073054	-0,026767	0,081623	0,185469	0,178960
1LV MM	-0,187984	-0,096165	-0,149325	-0,031387	0,225129	0,242149
1MMI, g/m2	-0,137707	0,001539	-0,168994	0,003509	0,193927	0,232872
1MMIh, g/m2,7	-0,309488	0,020763	-0,336208	0,107569	0,173316	0,307069
1LV EF	-0,341826	-0,123353	-0,243688	0,089642	0,102135	0,158756
1LV MWS	0,027230	-0,092224	0,135494	-0,015734	-0,013880	-0,060971

Supplementary table 1 (continued).

Variable	16MWD	16MWD%	26MWD	26MWD%	D.6MWD	D.6MWD%
1SI	0,048519	-0,027257	0,105526	-0,036523	0,029485	-0,011570
1RV	-0,032712	0,136098	-0,104937	0,181812	-0,013662	0,056277
1TAPSE	-0,220522	0,091782	-0,205313	0,139135	0,060196	0,126322
1RVLS	-0,296580	0,176210	-0,318509	0,233453	0,053332	0,190799
1RA	0,131352	0,123994	0,095844	0,106916	-0,105759	-0,089238
1LV E	-0,028490	-0,173652	-0,085724	-0,152796	-0,080586	-0,112201
1LV A	-0,344728	0,110402	-0,406039	0,237348	0,036737	0,145319
1LV E/A	0,217673	-0,241077	0,212545	-0,330321	-0,091085	-0,200498
1LV DTE	0,165198	0,538838	0,296866	0,769916	0,609994	0,424627
1LV s'	0,176320	-0,153828	0,244785	-0,122277	0,092342	-0,039229
1LV e'	0,288286	-0,275784	0,433459	-0,229795	0,030086	-0,154227
1LV a'	0,028181	0,236434	-0,037728	0,351817	0,034786	0,113166
1LV E'/A'	0,224500	-0,390021	0,367981	-0,414914	0,019611	-0,191558
1LV E/e'	-0,366414	0,093678	-0,562398	0,086299	-0,092800	0,066696
1LV E/e's'	-0,346385	0,113953	-0,474765	0,108447	-0,087892	0,069242
1LV E/GLS'	0,026045	-0,142422	-0,086818	-0,229761	-0,230198	-0,260105
2LA	-0,082748	-0,005766	-0,019986	0,027724	0,089771	0,124116
2LAVI	-0,246092	-0,036966	-0,217728	0,069567	0,087723	0,149625
2IVS	-0,434860	-0,164902	-0,402120	-0,044409	0,089026	0,170203
2LVPW	-0,389770	-0,061780	-0,334519	0,078637	0,115951	0,218891
2LV RWT	-0,345956	-0,001065	-0,362339	0,098118	0,021869	0,136112
2LV EDD	0,044112	-0,224531	0,151174	-0,216451	0,127158	0,018351
2LV ESD	0,138277	-0,159150	0,214328	-0,212827	0,088000	-0,019608
2MAPSEmed	0,048069	-0,144733	0,183087	0,028998	0,214245	0,172482
2MAPSElat	0,125222	-0,234628	0,221659	-0,081990	0,161911	0,067265
2LV GLS	-0,005728	-0,196176	0,087310	-0,079885	0,129548	0,088904

Supplementary table 1 (continued).

Variable	16MWD	16MWD%	26MWD	26MWD%	D.6MWD	D.6MWD%
2LV MM	-0,314168	-0,229074	-0,203793	-0,114123	0,164056	0,175451
2MMI, g/m2	-0,345987	-0,222664	-0,258520	-0,110678	0,120698	0,140974
2MMIh, g/m2,7	-0,486383	-0,126055	-0,415148	0,020175	0,099805	0,201035
2LV EF	-0,194235	0,020289	-0,213336	0,130890	-0,016358	0,050390
2LV MWS	0,123729	-0,043061	0,141957	-0,014416	0,012651	-0,054103
2SI	0,049682	-0,166626	0,101457	-0,137577	0,043788	-0,052662
2RV	0,044007	0,205187	0,019358	0,195319	0,057661	0,089538
2TAPSE	-0,008658	0,215501	-0,059177	0,203084	-0,029381	0,030327
2RVLS	-0,120377	0,322764	-0,237304	0,301390	-0,117082	0,003514
2RA	0,054905	-0,098975	0,058369	-0,111972	0,029363	-0,025076
2LV E	-0,199108	-0,340743	-0,199962	-0,344081	-0,101239	-0,129503
2LV A	-0,432534	0,100872	-0,498270	0,197334	-0,030393	0,108907
2LV E/A	0,135765	-0,392706	0,188312	-0,455847	-0,055777	-0,188277
2LV s'	0,302279	-0,102061	0,349043	-0,035673	0,072113	-0,023273
2LV e'	0,331973	-0,293935	0,410725	-0,320872	0,025787	-0,148251
2LV a'	0,310333	0,471249	0,200191	0,414822	-0,108162	-0,077597
2LV e'/a'	0,158632	-0,478314	0,262368	-0,492186	0,061412	-0,109110
2LV E/e'	-0,529126	-0,038587	-0,604508	-0,023039	-0,103299	0,022699
2LV E/e's'	-0,446330	-0,083548	-0,500807	-0,100373	-0,082630	-0,004107
2LV E/GLS	-0,207876	-0,283936	-0,252460	-0,345619	-0,179945	-0,198740
D.LA	0,043324	-0,157953	0,166316	-0,098611	0,157538	0,104892
D.LAVI	-0,122651	-0,304602	-0,012026	-0,186823	0,159116	0,140072
D.IVS	0,010123	-0,097855	-0,047435	-0,169178	-0,303205	-0,306417
D.LVPW	0,069762	0,168593	0,034144	0,103050	-0,185912	-0,131759
D.LV RWT	0,086580	0,119680	0,086851	0,042644	-0,165210	-0,134974
D.LV EDD	-0,111866	-0,280640	-0,104687	-0,180751	0,115469	0,053340

Supplementary table 1 (continued).

Variable	16MWD	16MWD%	26MWD	26MWD%	D.6MWD	D.6MWD%
D.LV ESD	-0,230284	-0,235768	-0,101419	-0,161628	0,204834	0,155795
D.MAPSEmed	0,040900	-0,030845	-0,030030	-0,113568	-0,112024	-0,119928
D.MAPSElat	0,151217	-0,085876	0,089491	-0,112779	-0,132175	-0,155694
D.LV GLS	0,118493	-0,057748	0,094745	-0,151610	-0,082143	-0,109077
D.LV MM	-0,046425	-0,175314	-0,107662	-0,175009	-0,145161	-0,158351
D.MMI, g/m2	-0,028916	-0,179917	-0,094396	-0,174808	-0,159865	-0,198128
D.MMIh, g/m2,7	-0,006477	-0,172099	-0,095841	-0,202332	-0,294162	-0,311073
D.LV EF	0,150215	0,055768	0,028442	0,040979	-0,125848	-0,118173
D.LV MWS	0,057805	-0,045418	-0,001716	0,001989	0,025318	0,007902
D.SI	0,030375	-0,168158	-0,026463	-0,078847	0,010959	-0,040077
D.RV	0,167853	0,048008	0,170156	0,039730	0,100168	0,055021
D.TAPSE	0,237910	0,125960	0,134385	0,036107	-0,092125	-0,087735
D.RVLS	0,259322	0,199907	0,112554	0,027165	-0,159162	-0,180030
D.RA	-0,091151	-0,335887	-0,042051	-0,228707	0,146419	0,071515
D.LV s'	0,117909	0,075826	0,094768	0,114103	-0,033922	0,022991
D.LV e'	0,069520	-0,228705	0,091848	-0,212364	0,001844	-0,034754
D.2LV a'	0,262514	0,135805	0,210863	0,029851	-0,121604	-0,165203
D.2LV e'/a'	-0,143287	-0,297710	-0,095512	-0,203374	0,071587	0,093648
D.2LV E/e'	-0,183729	-0,074598	-0,214662	-0,142210	-0,043142	-0,049504
D.2LV E/e's'	-0,254850	-0,150534	-0,283334	-0,196950	-0,041801	-0,052139
D.2LV E/GLS	-0,155928	-0,169814	-0,126455	-0,112600	0,063617	0,066947

Note. 1* - Visit 1; 2* - Visit 2; D.* - increment between visits. Correlations with P < 0,05 are marked in red. 6MWD – 6-minute walk distance, 6MWD% – reached percent of predicted 6MWD, LA – left atrium, LAVI – LA volume index, IVS – interventricular septum, LV – left ventricle, LVPW – LV posterior wall, RWT – relative walls thickness, EDD – end-diastolic diameter, ESD – end-systolic diameter, MAPSE – mitral annular plane systolic excursion, GLS – global longitudinal strain, MM – myocardial mass, MMI – myocardial mass index, EF – ejection fraction, MWS – midwall shortening, SI – stroke index, RV – right ventricle, TAPSE - tricuspid annular plane systolic excursion, RVLS – RV free wall strain, RA – right atrium.

Supplementary table 2. Left ventricular morphology in the study group and control vs external reference values (1).

Parameters	Control group, Sechi et al. (reference values)	COVID-19 General (2-sided p)	Control 1 (2-sided p)
Subjects, n	105	176	88
Hypertension prevalence	31 (30)	0,109	0,237
Interventricular septum, mm	9,0 ± 1,8	< 0,001	0,643
LV posterior wall, mm	8,9 ± 1,6	< 0,001	0,574
LV relative wall thickness	0,369 ± 0,072	< 0,001	0,034
LV end-diastolic diameter, mm	49 ± 5	< 0,001	0,001
LV mass index (height^{2,7}), g/m^{2,7}	35,5 ± 10,6	0,031	0,215

Note. T-test for independent samples for continuous variables, Chi-squared test for counted variables.

References

1. Sechi LA, Colussi G, Bulfone L et al. Short-term cardiac outcome in survivors of COVID-19: a systematic study after hospital discharge. Clin Res Cardiol 2021;110:1063-1072.
